# Biomarkers in Alzheimer Disease and Other Dementias: What’s Next into Pathophysiology to Support Clinical Practice and Drug Development

**DOI:** 10.3390/medicina58101374

**Published:** 2022-09-30

**Authors:** Anastasia Bougea

**Affiliations:** 1st Department of Neurology, Eginition Hospital, Medical School, National and Kapodistrian University of Athens, 72-74 Vassilisis Sofia’s Avenue, 11528 Athens, Greece; abougea@med.uoa.gr or annita139@yahoo.gr; Tel./Fax: +30-21-0728-9285

Neurodegenerative diseases are a heterogeneous group of disorders characterized by gradual progressive neuronal loss in the central nervous system [1]. These include Alzheimer’sdisease (AD), Parkinson’s disease (PD) with or without dementia, dementia with Lewy bodies (DLB), multiple system atrophy (MSA), progressive supranuclear palsy (PSP) and prion diseases. Unfortunately, the currently available therapies are limited to stabilization, ameliorating symptoms and slowing symptomatic progression, and have no clear effects on the mechanisms of progression [1]. This illustrates the need for the development of pathologically sensitive and easy-to-measure disease biomarkers to provide accurate diagnosis, prognosis andmonitoring of disease progression, as well asdrug efficacy inclinical trials, for future personalized medicine and prevention strategies. To date, studies ofprotein biomarkers (α-synuclein, Aβ42, Tau and pTau 181) in the cerebrospinal fluid (CSF) and the blood of these patients have shown conflicting results [2,3].

This Special Issue of Medicina, entitled “Biomarkers in Alzheimer Disease and Other Dementias: What’s Next into Pathophysiology to Support Clinical Practice and Drug Development”, includes seven articles dealing with the role of CSF plasma/serum biomarkers in the understanding the biochemical mechanisms and (differential) diagnosis of dementing disorders, and their drug development.

The CSF AD profile (increased levels of pTau and total tTau, and decreased levels ofAβ42) is highly predictive of the progression of cognitively unimpaired subjects to mild cognitive impairment (MCI) and dementia. In their interesting study, Scarmeas et al. [4] suggested that the AD continuum (either Alzheimer’s pathologic change or AD) and CSF profile were more likely to deteriorate cognitively over time. Another important finding of this study was that ApoE carrier levels were higher within the AD continuum group. These preliminary data highlight the importance of CSF biomarkers in determining cognitive decline in cognitively unimpaired subjects and individuals with MCI. They also demonstrate, once more, the underlying heterogeneity of patients, both in terms of CSF biomarker profiles and the future clinical course.

Using ultrasensitive single-molecule array assays (Simoa), it is possible to measure low concentrations of NfL, not only in the CSF but also in blood samples, with very high sensitivity. In this prospective study of patients with AD conducted by Kern et al. [5], higher sNfL concentrations were associated with worse cognitive performance, and there were no significant associations with respect to changes in neuropsychiatric symptoms and volumes of the brain regions typically affected by AD pathology.

In the future, biomarkers should be available not only for early diagnosis, but also for indicating responses to specific therapeutic interventions that help to guide treatment decisionsfor AD. Alharbi et al. [6] demonstrated the anti-inflammatory and anti-oxidant action of rosinidin in streptozotocin-induced rats, improving their cognitive and spatial learning behavior. These are promising results and suggest rosinidinas a therapeutical agent for further neurodegenerative disorder-related clinical trials.

Prion diseases are rare, irreversible neurodegenerative disorders with rapidly progressive dementia, cerebellar ataxia and myoclonus. Altuna et al. [7] reviewed current neuroimaging, neurophysiological and CSF biomarkers (14-3-3 protein and real-time quaking-induced conversion (RT-QuIC)). The sensitivity and specificity of some of these tests (electroencephalogram and 14-3-3 protein) areunder debate, and the applicability of other tests, such as RT-QuIC, is not universal. However, Altuna et al. [7] doubted the usefulness of these biomarkers beyond the most frequent prion disease, sporadic Creutzfeldt–Jakob disease, and further investigations are warranted.

Continuous efforts will be also necessary to establish useful CSF biomarkers for the early diagnosis of dementia with Lewy bodies (DLB). In their review, Foska et al. [8] highlighted the need foranother more indicative biomarker than classical AD biomarkers for DLB. They also emphasized the need for thecontrol of several confounding parameters, in addition to standardization of the *α*-syn species measured for further validation of these biomarkers in clinical practice.

Compared to protein-based biomarkers, miRNAs are more stable inbodily fluids; moreover, their easy measurement usingvarious commonly used laboratory methods make them potential biomarkers for atypical dementing disorders such asmultiple system atrophy (MSA) and progressive supranuclear palsy (PSP). In our systematic review [9], three studies have shown that miR-9-3p, miR-19a, miR-19b and miR-24 are potential biomarkers for MSA. In two studies, miR-132 was downregulated in the brain tissue of PSP patients, whereas miR-147a and miR-518e were upregulated. Pre-analytical and analytical factors of the included studies were important limitations to justifying the introduction of miRNAsfor MSA and PSP into clinical practice. More longitudinal studies should focus on whether there is a specific microRNA molecular fingerprint for each neurodegenerative disease.

Potagas et al. [10] suggested that speech-derived indices, and especially silent pauses, could be used as complementary biomarkers to efficiently discriminate between patients with Primary Progressive Aphasia (PPA) and healthy speakers, as well as between the three variants of the disease. These are important results that suggest the incorporation of such novel biomarkers into the diagnostic strategy of PPA, not only as classifiers, but also as quantified markers to clarify more the syndrome’s phenotypes.

Overall, the papers published in this Special Issue contribute to a deeper understanding of biochemical markers in dementing disorders, including neurodegenerative proteinopathies with novel biomarkers. Thereby, this Special Issue might initiate hypothesis-driven refinement of current and novel biomarkers in AD and other dementias.

## Data Availability

Not applicable.
